# Low-Dose Occupational Exposure to Ionizing Radiation and Cardiovascular Effects: A Narrative Review

**DOI:** 10.3390/healthcare12020238

**Published:** 2024-01-18

**Authors:** Guglielmo Manenti, Luca Coppeta, Ivan Valentinov Kirev, Greta Verno, Francesco Garaci, Andrea Magrini, Roberto Floris

**Affiliations:** 1 Department of Diagnostic and Interventional Radiology, Molecular Imaging and Radiotherapy, PTV Foundation, University of Rome Tor Vergata, 00133 Rome, Italy; 2 Department of Biomedicine and Prevention, University of Rome Tor Vergata, 00133 Rome, Italy; luca.coppeta@uniroma2.it (L.C.); greta.verno@students.uniroma2.eu (G.V.);

**Keywords:** cardiovascular disease, ionizing radiation, low-dose radiations, occupational exposure

## Abstract

Historically, non-cancer diseases have not been considered a health risk following low-dose exposure to ionizing radiation. However, it is now well known that high-dose ionizing radiation causes cardiovascular disease, and emerging epidemiological evidence suggests an excess risk of non-cancer diseases even following exposure to lower doses of ionizing radiation than previously thought. In fact, the evidence is strongest for cardiovascular disease (CVD). The aim of this review was to report the most representative studies and data on the risk of CVD from low-dose radiation in people with occupational exposure. We reported the results of 27 articles selected from a database search of 1151 studies. The results show a complex evidence landscape on the relationship between radiation exposure and cardiovascular disease. In general, published papers show a positive association between ionizing radiation exposure and dermal microcirculation damage, ischemic heart disease, and cerebrovascular disease. Overall, they highlight the need for comprehensive and detailed research to clarify this relationship. Due to limited statistical power, the dose–risk relationship below 0.5 Gy is inconclusive, but if this relationship is found to have no threshold, it could have a significant impact on current estimates of health risks at low doses.

## 1. Introduction

Ionizing radiation is a broad term encompassing any radiation capable of ionizing atoms or molecules within the medium it passes through. This interaction with biological matter has the potential to inflict damage on cells and tissues [1]. Radiation exposure to the human organism can occur externally or internally through the ingestion, inhalation, cutaneous absorption, or injection of radionuclides. The impact of radiation is directly linked to the dose received by individual cells or organs, as well as the quality of the radiation [2].

Although the harmful effects of high-dose ionizing radiation are well known, there is a growing interest in understanding the potentially harmful effects of exposure to low and moderate doses of ionizing radiation, particularly with regard to cardiovascular risk. Indeed, the relationship between exposure to low doses of ionizing radiation and cardiovascular risk has been and continues to be the subject of numerous scientific studies and research [3]. There is evidence of an increased risk of cardiovascular disease at lower dose levels, less than 5 Gy, and with mean doses well below 0.5 Gy, according to the Life Span Study (LSS), among atomic bomb survivors. Despite the shape of the dose–response relationship being unclear, especially for doses below 0.5 Gy, there was no evidence of a significant non-linear radiation dose–response relationship for cardiovascular disease mortality in the LSS data. Most recently, though still debatable, research indicates that exposure to much reduced radiation doses and dose rates, particularly from occupational and medical diagnostic exposures, may be linked to an increased risk of cardiovascular diseases [4].

It is known that behind the mechanisms involved in ionizing radiation-induced cardiovascular toxicity, much of the early damage appears to be caused by acute and chronic inflammatory changes. This leads to vascular dysfunction, cardiac remodeling, and atherosclerosis [5].

This relationship is of particular importance given the growing exposure of the population to ionizing radiation from sources such as diagnostic X-rays, radiotherapy, professional activities, and air travel. However, the full picture of the effects on the cardiovascular system following exposure to low doses of ionizing radiation is still subject to debate and investigation [6]. According to the World Health Organization (WHO), CVDs are a major global health issue—the primary cause of death worldwide—so we decided to investigate the current literature to analyze the main findings about CVD following low-dose radiation exposure in order to highlight these detrimental effects and debate regarding preventive measures to be used in the field to reduce the incidence of these diseases among the working population.

This review aims to provide an updated and comprehensive overview of the current scientific evidence on the association between low-dose ionizing radiation exposure and cardiovascular risk in occupationally exposed individuals.

The main objective is to summarize existing knowledge, identify the most representative articles, highlight potential gaps in the literature, and provide directions for future research on this topic.

Understanding whether there is a threshold dose for the cardiovascular effects of ionizing radiation may enable the implementation of preventive programs that reduce operator exposure to values below the threshold dose, thereby eliminating the risk of cardiovascular accidents.

## 2. Materials and Methods

### 2.1. Research Questions

The main purpose of this narrative review is to scrutinize the existing literature regarding the risk of cardiovascular disease in individuals exposed to low doses of ionizing radiation for occupational reasons. We narrowed the scope of our research by excluding subjects exposed to high doses and those undergoing radiotherapeutic treatments. We also want to identify deficiencies in the existing literature to propose directions for more focused and comprehensive future research. The search terms we used were occupational exposure, cardiovascular disease and risk, low-dose radiation, and ionizing radiation.

### 2.2. Study Selection and Data Items

The study focused on a population primarily composed of employed individuals regularly exposed to ionizing radiation.

We systematically searched the literature in September 2023 using PubMed with no restrictions (date, language).

### 2.3. Eligibility Criteria

The database search conducted by reviewers resulted in a total of 1151 articles. Initially, their eligibility was determined by evaluating just the abstract and title using the web tool Rayyan. More comprehensive information was used in the second stage of the search.

We excluded articles flagged as duplicates by Rayyan, articles based on in vitro or animal studies, articles not in English, all studies without an abstract, articles where reviewers did not reach an inclusion agreement, and those where the main population was individuals exposed to radiation for therapeutic purposes, such as patients undergoing radiotherapy.

**Figure 1:**
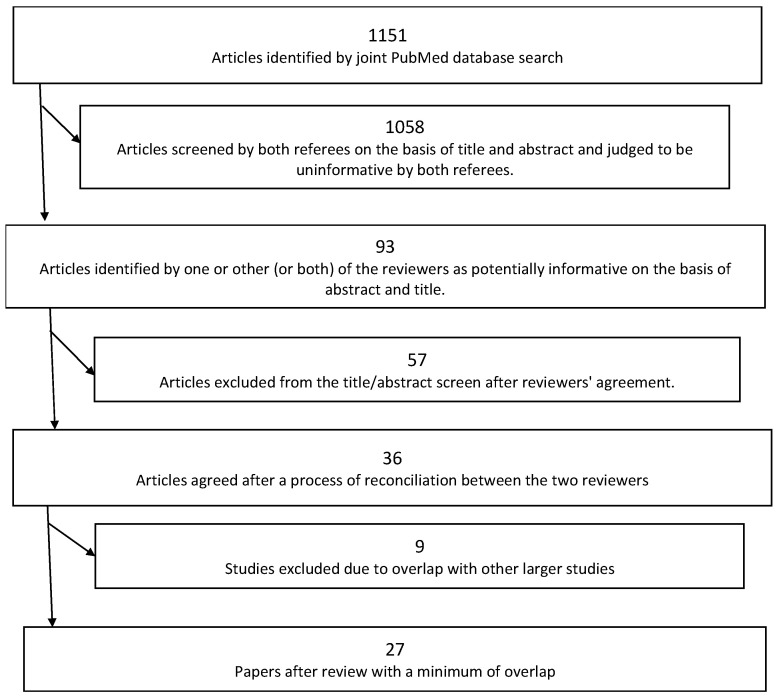
Flowchart showing exclusions made to derive the final set of studies used.

This resulted in a final set of 27 representative articles (Figure 1). Two reviewers independently extrapolated the information from the final articles using a spreadsheet and schematized the most relevant results.

## 3. Results

### 3.1. Radiation and Early Atherosclerosis

In 1993, Tomei et al. conducted a capillary microscopy study on 145 physicians exposed to occupational ionizing radiation, finding a strong association with dermal microcirculation damage. Only 20.7% of the exposed group had a normal capillaroscopic picture, compared to 91.5% in the control group. The relative risk ratio for capillaroscopic damage in exposed individuals was 0.23 (95% CI: 0.16–0.31). Interestingly, no notable variations were observed among medical specialties. This study highlights a compelling link between occupational radiation exposure and capillaroscopic abnormalities, with gender-specific nuances [7].

The correlation between low-dose ionizing radiation exposure and endothelial damage, specifically the risk of atherosclerosis, has been the subject of study by Andreassi et al. All participants were invited to undergo assessments, including carotid intima-media thickness (CIMT), peripheral blood testing for telomere length, and genetic evaluation. CIMT assessments were conducted on 171 cath lab staff and 156 unexposed subjects. Upon categorizing the cath lab staff into low- (*n* = 80) and high-exposure (*n* = 91) workers based on the median of occupational radiation exposure, a notable finding emerged. CIMT exhibited a significant increase in the high-exposure workers compared to their low-exposure counterparts. Exposed workers also showed a significant telomere shortening. These results suggest evidence of accelerated vascular aging and early atherosclerosis [8].

### 3.2. Ischemic Heart Disease (IHD)

The initial indication suggesting a potential heightened risk of cardiovascular disease (CVD) at low or moderate radiation doses originated from an examination of Japanese atomic bomb survivors. Since then, numerous other cohorts, including radiation and nuclear workers, accident responders, and environmentally exposed populations, have been subjected to scientific scrutiny, yielding outcomes that vary and present a range of results in relation to the correlation between radiation exposure and the risk of cardiovascular diseases.

In fact, analysis of cardiac mortality in the LSS from 1950 to 2008 showed positive associations between radiation dose and mortality rates for total heart disease (ERR/Gy = 0.14, 95% CI: 0.06–0.22), valvular heart disease (ERR/Gy = 0.44; 95% CI: 0.13–0.85), hypertensive organ damage (ERR/Gy = 0.36, 95% CI: 0.10–0.68), and heart failure (ERR/Gy = 0.21, 95% CI: 0.07–0.37). The dose–response model showed no statistically significant deviation from linearity [9].

The INWORKS study is a comprehensive collaborative cohort investigation encompassing 308,297 nuclear industry personnel (average cumulative external exposure, 0.025 Gy) across the United Kingdom, France, and the United States. As a result of specifically analyzing non-cancer mortality, a statistically significant excess relative risk per Sv (ERR/Sv) of 0.19 (90% CI: 0.07–0.30) was found for all non-cancer diseases, primarily driven by circulatory diseases (ERR/Sv = 0.22; 90% CI: 0.08–0.37). Ischemic heart disease (IHD) had a significantly elevated ERR/Sv of 0.18 (90% CI: 0.004–0.36), particularly driven by acute myocardial infarction (MI) with an ERR/Sv of 0.26 (90% CI: 0.03, 0.51). The risk for chronic IHD showed little evidence of elevation (ERR/Sv = 0.07; 90% CI: −0.19, 0.36), with no significant heterogeneity between subtypes (*p* = 0.38). The ERR/Sv estimate for other heart diseases (non-IHD) was comparable to IHD [10,11].

Laurent et al. analyzed a cohort of 22,393 employees of a French electricity company. They observed no significant increase in the RR/100 mSv (10 rem) of mortality due to circulatory disease or ischemic heart disease. There was an increased risk of cerebrovascular disease based on only 22 cases (RR/100 mSv (10 rem) 2.74, 90% CI: 1.02–5.39) [12].

Mortality due to cardiovascular diseases was investigated in an expanded Mayak employee cohort, comprising 22,377 workers initially hired at the Mayak Production Association between 1948 and 1982 and followed up to 2018. Many of the workers received chronic exposures from gamma radiation and/or plutonium intake. Significant upward trends in ischemic heart disease incidence were evident in the initial follow up, both for total external gamma dose and internal liver dose. The correlation between external dose and mortality attributed to ischemic heart disease did not reach statistical significance. Significant upward trends in the incidence of cerebrovascular disease, though not in mortality, were noted for both the complete external gamma dose and internal liver dose. Workers with cumulative gamma doses exceeding 1 Gy exhibited heightened risks of morbidity associated with ischemic heart disease and cerebrovascular disease. The dose–response associations for external radiation and the incidence of circulatory diseases were indicative of linearity for ischemic heart disease (ERR/Gy = 0.11, 95% CI: 0.05–0.17) and cerebrovascular disease (ERR/Gy = 0.46, 95% CI: 0.36–0.57). A subsequent investigation into the incidence and mortality of ischemic heart disease (IHD) highlighted a statistically significant rising trend in total external gamma-ray dose and IHD incidence (ERR/Gy = 0.099, 95% CI: 0.045–0.153), following adjustments for various factors such as hypertension, body mass index, duration of employment, and internal exposure [13,14,15,16].

Relative risks and excess relative risks per unit absorbed dose (ERR/Gy) were computed for the whole Mayak cohort, the Ozyorsk resident’s sub-cohort, and the migrant sub-cohort in a recent study. For males and females, the mean cumulative liver absorbed gamma-ray doses from external exposure were 0.45 Gy and 0.37 Gy, respectively. For males and females, the mean cumulative liver-absorbed alpha doses from internal plutonium exposure were 0.18 Gy and 0.40 Gy, respectively. The linear model, adjusting for non-radiation factors and alpha radiation dose, showed no significant associations of mortality from CVD, IHD, and cerebrovascular disease (CeVD) with a gamma-ray exposure dose in the entire cohort, resident sub-cohort, or migrant sub-cohort. While there were no significant correlations detected between liver-absorbed gamma dose and other outcomes, a noteworthy connection between gamma dose and male ischemic stroke mortality was noted in the resident sub-cohort (ERR/Gy = 0.43, 95% CI: 0.08–0.99). Men did not significantly correlate the liver absorbed alpha dose with mortality from any CVD when it came to internal exposure, but women did demonstrate positive correlations with mortality from IHD (whole cohort) and CVD (entire cohort and resident sub-cohort) [15].

In a recent article, Wakeford et al. reported the latest updates from the extensive studies conducted by Azizova et al. in which, after adjusting for non-radiation factors, a noteworthy upward trend was identified in circulatory disease mortality concerning increasing doses from external gamma-rays, yielding ERR/Gy of 0.05 (95% confidence interval (CI): >0–0.11). The inclusion of an additional adjustment for dose from internal alpha-radiation to the liver resulted in a twofold increase in ERR/Gy to 0.10 (95% CI: 0.02–0.21) [17].

Ivanov et al. (2001) [18] have analyzed the mortality of emergency workers at the Chernobyl accident who are living in Russia. Their study was conducted on data from the cohort of 65,905 emergency workers, which included reported external exposures of 0.005–0.3 Sv. There were discovered to be statistically significant risks for cardiovascular disease and malignant neoplasm mortality. In particular, the values of ERR/Sv^−1^ for cardiovascular diseases are estimated to be 0.54 (95% CI: 0.18–0.91) for the external control, which corresponds to the mortality rate (males) for the same age in Russia in general, and 0.79 (95% CI: 0.07–1.64) for the internal control. However, the mortality rate in the EMS cohort is increasingly approaching that of the general Russian population [18].

In addition, an analysis of the incidence of CeVD and CVD in the same cohort of Russian workers involved in the Chernobyl recovery operations is presented in several studies. The liquidators (53,772 persons) who reached the Chernobyl zone within the first year following the catastrophe (from April 1986 to April 1987) compose the cohort under study. In the cohort, the average external whole-body dosage was 0.161 Gy, with individual doses ranging from 0.0001 Gy to 1.42 Gy. A statistically significant dosage response was seen in the incidence of CeVD, with no latency time and an average ERR/Gy = 0.45, 95% CI: (0.28–0.62), *p* < 0.001. The length of the liquidators’ stay in the Chernobyl zone showed a statistically substantial correlation (*p* = 0.03) with the radiation risks of CVD. For those who spent less than six weeks in the Chernobyl zone, the ERR/Gy = 0.64, 95% CI = (0.38–0.93), *p* < 0.001. The incidence of CVD showed a statistically significant dose response with no latency period and an average ERR Gy = 0.47, 95% CI = 0.31–0.63, *p* < 0.001. The radiation risks of CVD showed a statistically significant variation (*p* = 0.01) with the length of time spent in the Chernobyl zone by liquidators. For those who spent less than six weeks in the Chernobyl zone, ERR/Gy = 0.80, 95% CI = 0.53–1.08, *p* < 0.001 [19,20].

A study of circulatory disease risks in a cohort of 337,397 workers included in the Canadian National Dose Registry reports a significant dose–response relationship was observed for CVD, with an excess relative risk (ERR) of 1.22 (90% CI: 0.47–2.10) for men and 7.4 (90% CI: 0.95–18.1) for women. Men had a mean total body radiation dose of 8.6 mSv, and women 1.2 mSv. The absolute excess risk for the entire cohort was 37.5 per sievert per 10,000 person-years (90% CI: 17.0–60.1), which suggests a positive association between radiation exposure and CVD mortality [21].

### 3.3. Cardiac and Cerebral Ischemic Mortality in Uranium Enrichment Workers

A strong positive and statistically significant correlation was found between radiation exposure and deaths from arteriosclerotic heart disease, including coronary heart disease, in a cohort of 53,698 workers employed in 15 nuclear power generating utilities in the United States (monitored for a maximum period of 18 years, from 1979 to 1997). The correlation had an ERR of 8.78 (95% CI 2.10–20.0). For the entire group, the mean total cumulative equivalent dosage was 25.7 mSv, and for those who had some documented exposure, it was 30.7 mSv. Although several earlier occupational studies have found connections with heart disease, the current association’s magnitude is inconsistent with these relationships, requiring a careful interpretation and more investigation [22]. In a previously studied cohort of US uranium enrichment workers, Anderson et al. examined the dose–response associations between mortality from IHD and CeVD and the absorbed ionizing radiation dose to the lungs from internal exposure to uranium and external ionizing radiation. The full cohort consisted of 29,303 workers. Their most significant findings indicate that subjects with an internal lung dose of uranium greater than 1 milligray (mGy) showed a slightly elevated hint of IHD risk (RR = 1.4; 95% CI: 0.76–2.3) and the linear excess relative rate (ERR) per mGy in full cohort of 0.019 (95% CI: −0.077–0.26) [23].

Another paper examines circulatory system disease mortality after long-term exposure to uranium in a sample of 2897 workers employed at a uranium processing plant in France during the period 1960–2006. They reported that circulatory system disease (CSD) mortality was increased in workers exposed to slow-soluble reprocessed uranium (HR = 2.13, 95% CI = 0.96–4.70) and in workers exposed to natural uranium (HR = 1.73, 95% CI = 1.11–2.69) [24].

Later, Zhivin et al. suggested conducting nested case–control research on nuclear workers in France. There were 102 people in the case group and 416 people in the control group. The maximum dose of 27 mGy was reported for the lung, while the calculated mean cumulative internal doses from uranium intakes varied from 0.01 mGy (heart) to 1 mGy (lung). Even after adjusting for uranium dose, there was no significant correlation found between CSD mortality and external γ-radiation exposure (at 1 mGy). The outcomes for CeVD and IHD were comparable. All cumulative internal uranium dosage categories showed an increased risk of CSD death, however, the test for departure from a trend was not statistically significant (*p* = 0.4). After adjusting for the most significant individual risk variables for CSD, their analyses revealed no indication of confounding and a positive, albeit imprecise, correlation between cumulative internal uranium dosage and CSD mortality [25].

Drubay et al. include risk factors for systemic circulatory disease in their analysis of the association between radiation exposure and mortality risk for CSD in a case–control nested study of uranium miners. In the entire cohort (5086 miners), cumulative radon exposure was significantly associated with an increased risk of death from CSD and CeVD hazard ratios: HR (CSD/100) working level months (WLM) = 1.11, 95% confidence interval (1.01–1.22) and HRCeVD/100 WLM = 1.25 (1.09–1.43), respectively [26].

Baseline results from a study of 20,608 Korean workers exposed at work revealed that hyperlipidemia and cardiovascular disease were the most prevalent non-cancer diseases in the cohort, however, no correlation was observed between these conditions and occupational exposure. The cohort’s mean cumulative exposure during the work period of 1984 to 2017 was 11.8 (standard deviation [SD] 28.8) mSv, whereas the median was 0.6 mSv. Age- and sex-specific standardized prevalence ratios (SPRs) and 95% Cis comparing the prevalence of cardiovascular disease in radiation workers with that in the general population were 0.95 (95% CI: 0.90–0.99) and 0.88 (95% CI: 0.68–1.11) in men and women, respectively. In the univariate analysis evaluating the association between radiation dose and the occurrence of various non-cancer diseases, higher prevalence rates were observed with higher cumulative doses. However, these associations did not reach statistical significance after adjustment for confounders [27].

In a recent study assessing 53,860 male medical radiation personnel who were registered in the National Dosimetry Registry (NDR) in South Korea (from 1996 to 2011), an estimated ERR per 100 mGy for CD [0. 85, 95% CI −0.11–1.82], ischemic heart disease (1.18, 95% CI −0.69–3.05), and cerebrovascular disease (0.23, 95% CI −0.48–0.94) with a 10-year lag was obtained using a linear dose–response model. The study found no statistical evidence of a dose–response relationship. The personnel who survived and those who died from CD had mean cumulative badge doses of 27.1 mSv and 10.2 mSv, respectively [28,29]. Engels et al. investigated cause-specific mortality among nuclear operators from five nuclear sites in Belgium, comparing the results with those of the general population. The study included a total of 7229 workers employed between 1953 and 1994, with the majority receiving less than 10 mSv of occupational exposure. The analysis focused on cause-specific mortality for both male and female workers, examining specific cancers, cardiovascular diseases, and respiratory diseases as endpoints. Importantly, no significant increase in mortality was identified in the study [30].

Frank de Vocht conducted an analysis using data from a matched nested case–control study within a cohort of British male industrial workers involved in the nuclear fuel cycle. This population comprised 1220 matched male case–control pairs. Cumulative external radiation doses varied from 0 to 1656 mSv, while cumulative internal doses, monitored for radioactive intakes, spanned from 0.004 to 5732 mSv. The risk of IHD mortality was found to be associated with cumulative unlagged external dose, revealing an excess risk of 42% (95% CI: 4%, 95%) for doses exceeding 103 mSv (highest quartile relative to the lowest quartile) and 35% (95% CI: −1%, 84%) for doses exceeding 109 mSv with a 15-year lagged dose [31]. Potential confounding variables like blood pressure, BMI, occupational exposure, socioeconomic status, smoking, and internal radiation monitoring are considered in a later study. The research indicates a non-linear dose–response relationship of 43% increased risk at 390 mSv by quantitative bias studies and simulations. After accounting for potential confounding, missing data, and several scenarios of exposure measurement error, it concludes that the observed connection between external radiation exposure and IHD mortality may be causative [32].

In a study of a sub-group (*n* = 4054) of the WISMUT cohort, Kreuzer et al. analyzed data for men who worked in uranium mills between 1946 and 1989. Workers in underground or open-pit mines were not considered. No increase in mortality risk with increasing cumulative dose was found for any cardiovascular disease (ERR/Sv −0.09; 95% CI: −1.04–0.86) or ischemic heart disease (ERR/Sv −0.10, 95% CI: −1.48–1.27). There was an observed statistically non-significant rise (ERR/Sv 0.55, 95% CI: −1.72–2.83) in mortality related to cerebrovascular disease [33].

### 3.4. Diagnostically Exposed Groups

The goal of the U.S. Radiologic Technologists (USRT) study was to calculate the incidence and mortality risks of cancer and cardiovascular disease related to the use of radionuclides during operations. They determined multivariable-adjusted hazard ratios (HRs) and 95% confidence intervals for the incidence (up to 2003–2005) and mortality (up to 2008) associated with conducting these operations from a national cohort of 90,955 US radiologic technologists. Analysis of cardiovascular disease-related mortality was limited to 86,700 technicians. They found that undergoing brachytherapy operations was linked to increased rates of all-cause death (HR = 1.10, 95% CI: 1.00–1.20), mortality from all malignancies (HR = 1.20, 95% CI: 1.01–1.43), and myocardial infarction (HR = 1.37, 95% CI: 1.10–1.70) [34].

The main results are summarized in Table 1.

## 4. Discussion

The studies reviewed present a complex landscape of findings regarding the relationship between radiation exposure and cardiovascular diseases. They collectively underscore the need for comprehensive and nuanced research to elucidate this relationship. One commonality among these studies is the focus on populations exposed to ionizing radiation, whether through occupational settings, medical procedures, or environmental incidents. This shared element highlights the importance of understanding the potential health consequences of radiation exposure given its widespread use and the ubiquity of background radiation.

However, the differences among the studies are equally notable. These disparities arise from variations in study populations, radiation dose levels, specific cardiovascular disease endpoints, study designs, geographical factors, and the extent of adjustments for potential confounding variables. These divergences complicate the interpretation of results and highlight the multifaceted nature of the relationship between radiation and cardiovascular health. The presence of mixed findings in these studies is a critical observation. For instance, some studies suggest a heightened risk of cardiovascular diseases, particularly ischemic heart disease, associated with radiation exposure. The variation in radiation dose levels across studies is of particular interest. Some studies involve individuals exposed to relatively low doses, which is relevant to medical diagnostic and occupational radiation exposure scenarios. For example, in the study by Soojin Park et al., the mean cumulative dose in the cohort was 11.8 mSv.

Others include individuals with higher cumulative doses, such as nuclear industry personnel and Chernobyl emergency workers. Additionally, the differences in study designs, including cohort studies, case–control studies, and nested case–control studies, introduce methodological variations that affect the interpretation of findings. For example, Kreuzer et al. conducted a nested case–control study of uranium miners and reported a statistically non-significant rise (ERR/Sv 0.55) in mortality related to cerebrovascular disease. The geographical variations among these studies introduce a crucial aspect of real-world diversity. Different healthcare systems, lifestyles, and environmental factors in various countries can influence cardiovascular disease risk independently of radiation exposure. For instance, the INWORKS study, which encompassed 308,297 nuclear industry personnel across France, the United Kingdom, and the United States, found a statistically significant excess relative risk per Sv (ERR/Sv) of 0.19 for all non-cancer diseases, primarily driven by circulatory diseases (ERR/Sv = 0.22) and ischemic heart disease (ERR/Sv = 0.18).

Little et al. coordinated several groups that have published important comprehensive reviews as well as several meta-analyses of the available epidemiological evidence. A past meta-analysis of epidemiological data on the incidence of cardiovascular disease after low to intermediate doses of radiation (mean heart/brain doses all <2.5 Sv and mostly <0.5 Sv) suggested positive and significant aggregated ERR/Sv for CVD of 0.08 (95% CI 0.05–0. 11), heart disease 0.07 (95% CI 0.04–0.11), and stroke 0.27 (95% CI 0.20–0.34) [4]. A more recent meta-analysis showed that radiation exposure was associated with a generally significant meta-excess relative risk per Gy for total cardiovascular disease 0.11 (95% confidence interval 0.08–0.14), ischemic heart disease 0.07 (0.05–0.10), other heart disease 0.03 (0.02–0.05), cerebrovascular disease 0.19 (0.09–0.28), and other cardiovascular disease 0.17 (−0.03–0.37) [35].

Adjustment for potential confounding factors is another crucial consideration. Some studies meticulously account for variables such as smoking, BMI, and socioeconomic status, while others may have more limited adjustments. These adjustments play a pivotal role in ensuring that observed associations are not confounded by external factors. For instance, the USRT study reported that performing brachytherapy procedures was associated with higher incidence of myocardial infarction (HR = 1.37) and all-cause mortality (HR = 1.10) after adjusting for relevant factors. Caution must be exercised in interpreting these results because of the potential bias introduced by dosimetry uncertainties, possible errors in record linkage, and especially the lack of adjustment for non-radiation risk factors. The main limitation of our review is that we could not take into account all of the potential confounding factors present in the studies reviewed—in particular, lifestyle factors, the presence of genetic predisposition to the development of CVD, geographical factors, and the type of population studied (healthcare workers, nuclear accident survivors, and nuclear industry workers). Further studies may be useful to assess the effect of all these factors on the risk of CVDs. Another limitation is that we did not include papers written in languages other than English.

Based on our review, it is crucial to minimize exposure to ionizing radiation.

In Italy, Legislative Decree 101/2020 regulates the dose values that must be respected to avoid deterministic effects of ionizing radiation and reduce the incidence of probabilistic ones in the workplace.

Respecting these limits can also reduce the risk of CVD. However, the best preventive measure is to keep ionizing radiation levels as low as possible to achieve the desired objectives [36].

## 5. Conclusions

Regarding radiation protection, the scientific topic of whether long-term exposure to low doses or low-dose rates of radiation from external sources increases the risk of cardiovascular disease is still crucial. Although they are still prone to bias, observational epidemiological studies in communities of radiation workers across several nations offer compelling data. Because the impact of other environmental, lifestyle, and personal risk factors outweigh the risk of cardiovascular disease from low-dose radiation exposure, an epidemiological study’s statistical power is crucial. A lack of information on lifestyle factors, especially those that affect etiology and include alcohol intake, smoking, food, obesity, physical activity, and genetic background, is a general limitation of the low-dose investigations of CVD that are now being conducted. The two key recommendations for future research are to gather more biological samples in order to better understand the effect of radiation on the disease process and to carry out large adult cohort studies involving Japanese atomic bomb survivors, laborers, and clinical trials.

## Figures and Tables

**Table 1:** Summary of selected studies and main results.

Section	Study ID	Study Type	Study Design	Main Results
**Early atherosclerosis**	Tomei, F. et al. 1996 [7]	Comparative study	The aim of the study is to use capillary microscopy to investigate damage to the dermal microcirculation caused by occupational exposure to ionizing radiation doses below 5 rem/year, focusing on a sample of 145 physicians.	The study shows that low doses of ionizing radiation can cause morphological changes in capillaries, as observed by capillaroscopy. These changes, which occur before clinical signs, may serve as early indicators of vascular damage.
	Andreassi, M.G. et al. 2015 [8]	Comparative study	The study aims to link prolonged radiation exposure in the cath lab with early indicators of subclinical atherosclerosis. It will measure carotid intima-media thickness in 223 cath lab workers compared to 222 unexposed subjects.	Prolonged exposure to radiation in the cath lab may correlate with increased subclinical carotid intima-media thickness and shortened telomere length, signs of accelerated vascular aging, and early-stage atherosclerosis.
**Ischemic Heart Disease**	Takahashi, I. et al. 2017 [9]	Epidemiological study	This study aims to investigate the cardiovascular risks associated with radiation at lower dose levels through subtype and period-specific analyses in a population of 86,600 individuals, including a large proportion of atomic bomb survivors (Life Span Study).	Although the radiation doses of the atomic bomb survivors were generally much lower than those in other studies that have shown an increased risk of vascular disease due to exposure to ionizing radiation, the current analysis shows a significantly increased risk of heart disease overall over the entire period.
	Gillies, M. et al. 2017 [10]	Epidemiological study	This study examines the associations between low-level exposure to ionizing radiation and non-cancer disease mortality were examined among nuclear industry workers from France, United Kingdom (UK), and United States (U.S.), as part of the International Nuclear Workers Study.	In the INWORKS cohort, elevated external radiation levels were significantly linked to non-cancer mortality. The increased risk predominantly stemmed from a linear relationship with circulatory diseases, specifically ischemic heart disease and cerebrovascular disease.
	Laurier, D. et al. 2017 [11]	Epidemiological study	This study presents a comparison of radiation cancer mortality risk estimates derived from the Life Span Study and the International Nuclear Workers Study, comparing a selection of 45,625 atomic bomb survivors and 259,350 nuclear workers.	In both cohorts, there was evidence of a modification of the excess absolute risk of solid cancers with age attained. These findings from different study populations may contribute to the understanding of radiation risks, with an important contribution coming from cohorts of workers with prolonged low-dose rate exposures.
	Laurent, O. et al. 2010 [12]	Epidemiological study	This study examined the relationships between exposure to ionizing radiation and mortality in workers of the French Electricity Company who were followed up from 1961 to 2003 (*n* = 22,393).	A total of 874 deaths occurred and 66 workers were lost to follow up. None of the causes of death studied increased significantly with dose except for cerebrovascular disease (*p* = 0.01), but this last observation was based on only 22 cases.
	Azizova, T.V. et al. 2012 [13]	Epidemiological study	Incidence and mortality from ischemic heart disease was studied in an extended cohort of 22,377 workers first employed at the Mayak Production Association during 1948–1982 and followed up to the end of 2008.	This research provides compelling evidence linking external gamma radiation exposure to the incidence and mortality of ischemic heart disease. In addition, there is some evidence of an association between internal alpha radiation exposure and the incidence and mortality from ischemic heart disease.
	Azizoza, T.V. et al. 2015 [14]	Epidemiological study	This study examined mortality from circulatory disease in an extended Mayak worker cohort of 22,377 workers first employed at the Mayak Production Association in 1948–1982 and followed up to the end of 2008.	Analyses of mortality from circulatory diseases among members of the Mayak worker cohort first recruited between 1948 and 1982 showed a significant increasing trend in mortality from circulatory diseases with dose from external gamma radiation.
	Azizoza, T.V. et al. 2022 [15]	Epidemiological study	This paper reports the results of the study of mortality from diseases of the circulatory system among Russian nuclear workers at the Mayak Production Association (22,377 individuals) hired at the plant between 1948 and 1982 and followed up until the end of 2018.	The analysis of mortality from circulatory diseases among members of the Mayak worker cohort showed a significant increasing trend in mortality from circulatory diseases with dose from external gamma radiation. Inclusion of adjustment for internal alpha dose to the liver resulted in a two-fold estimate of the ERR/Gy.
	Azizoza, T.V. et al. 2023 [16]	Epidemiological study	The objective of this study was to assess the incidence of various subtypes of heart diseases in a cohort of workers chronically exposed to ionizing radiation in relation to radiation dose and non-radiation factors.	In the present study, was found evidence for a significant positive association of certain types of heart diseases (ischemic heart disease, angina pectoris, heart failure, and cardiac arrhythmia and conduction disorder) among males and females with occupational chronic external exposure to ionizing radiation was found.
	Ivanov, V.K. et al. 2001 [18]	Epidemiological study	This paper presents the results of the analysis of mortality among Chernobyl emergency workers living in Russia, including 65,905 persons.	The dose response of mortality was investigated. Statistically significant radiation risks were found for mortality from malignant neoplasms (515 cases) and cardiovascular diseases (1728 cases).
	Kashcheev, V.V. et al. 2001 [19]	Epidemiological study	The study presents an analysis of the incidence of cerebrovascular diseases among Russian workers involved in recovery tasks after the Chernobyl accident, including 53,772 recovery operation workers.	Considering as risk factors for the incidence of cerebrovascular diseases: dose, duration of work of the liquidators in the Chernobyl area and concomitant diseases (hypertension, ischemic heart disease, atherosclerosis, and diabetes) a statistically significant association was seen towards all concomitant diseases studied. The incidence of cerebrovascular disease revealed a statistically significant dose response with the absence of a latency period.
	Kashcheev, V.V. et al. 2017 [20]	Epidemiological study	This study analyzes the impact of radiation on incidence of circulatory system diseases in the cohort of Russian recovery operation workers and presents the results of the analysis of cardiovascular disease incidence.	The incidence of cardiovascular disease has revealed a statistically significant dose response with the lack of a latent period. Radiation risks of cardiovascular disease showed statistically significant variation with the duration of liquidators’ stay in the Chernobyl zone; for those who stayed in the Chernobyl zone less than six weeks.
	Zielinski, J.M. et al. 2009 [21]	Cohort study	This study analyzes the risk of cardiovascular disease mortality in a Canadian cohort of 337,397 individuals occupationally exposed to ionizing radiation and included in the National Dose Registry of Canada.	The research has shown a significant correlation between the amount of radiation exposure and the likelihood of cardiovascular disease mortality.
**Cardiac and cerebral ischemic mortality**	Howe, G.R. et al. 2004 [22]	Cohort study	This study analyzes the mortality amongst U.S. nuclear power industry workers after chronic low-dose exposure to ionizing radiation. A total of 53,698 workers employed in 15 utilities that generate nuclear power in the United States have been followed for up to 18 years between 1979 and 1997.	A strong positive and statistically significant association between radiation dose and deaths from arteriosclerotic heart disease including coronary heart disease was observed in the cohort.
	Anderson, J.L. et al. 2021 [23]	Epidemiological study	In this study, linear and non-linear dose–response relationships between radiation absorbed dose to the lung from internally deposited uranium and external sources and circulatory system disease mortality were examined in a cohort of 23,731 male and 5552 female US uranium enrichment workers.	Non-linear dose–response models using restricted cubic splines revealed sublinear responses at lower internal doses, suggesting that linear models that are common in radioepidemiological cancer studies may poorly describe the association between uranium internal dose and cardiovascular disease mortality.
	Guseva Canu, I. et al. 2012 [24]	Cohort study	This work examines the circulatory system disease mortality after chronic intake of uranium among 2897 workers (79,892 person-years) at a uranium processing plant (1960–2006) in France.	The authors observed that exposure to slowly soluble uranium, namely reprocessed uranium, may increase the risk of circulatory system disease mortality.
	Zhivin, S. et al. 2018 [25]	Case-control study	This paper is a nested case–control study of French AREVA NC Pierrelatte nuclear workers employed between 1960 and 2005 to estimate circulatory system disease risks adjusting for major circulatory system disease risk factors (smoking, blood pressure, body mass index, total cholesterol, and glycaemia) and external γ-radiation dose.	This study suggests that a positive association might exist between internal uranium exposure and circulatory system disease mortality, not confounded by circulatory system diseases risk factors.
	Drubay, D. et al. 2015 [26]	Case-control study	This study analyses the French cohort of uranium miners (*n* = 5086), which included 442 deaths from circulatory system diseases, 167 of them from ischemic heart disease and 105 from cerebrovascular disease.	These results suggest that the significant relationship between cerebrovascular disease risk and radon exposure observed in the total French cohort is probably not affected by the circulatory system diseases risk factors.
	Park, S. et al. 2021 [27]	Epidemiological study	This study aims to evaluate the association between occupational radiation exposure and the prevalence of non-cancer diseases among 42,607 Korean radiation workers.	The baseline findings indicated that hyperlipidemia and diseases of the circulatory system were the most prevalent non-cancer diseases in the cohort; however, there was no association between hyperlipidemia or circulatory system diseases and occupational exposure.
	Cha, E.S. et al. 2020 [28]	Epidemiological study	This study investigates the association between low-dose external occupational radiation exposure and circulatory disease morbidity among diagnostic medical radiation workers (*n* = 11,500).	This study provides little evidence in support of a positive association between occupational radiation exposure and the overall risk of circulatory disease over a short follow-up period among medical radiation workers in South Korea.
	Bang, Y.J. et al. 2023 [29]	Epidemiological study	The aim of this study is to investigate the relationship between occupational radiation exposure and circulatory disease mortality among medical radiation workers (*n* = 53,860).	Occupational radiation doses were non-significantly positively associated with circulatory disease mortality among male diagnostic medical radiation workers
	Engels, H. et al. 2005 [30]	Epidemiological study	In this study cause-specific mortality was studied in nuclear workers from five nuclear facilities in Belgium and compared to the general population.	Analysis of cause-specific mortality was performed for male and female workers for three endpoints: specific cancer sites, cardiovascular diseases, and respiratory diseases. No significant increase in mortality was observed.
	de Vocht, F. et al. 2020 [31]	Comparative study	This study is a matched case–control study of male industrial workers employed at one of two nuclear installations formerly operated by BNFL, involved in production and manual skilled and unskilled work associated with operating and maintaining nuclear fuel cycle plants.	This study confirms associations between cumulative radiation doses from external sources and mortality from ischemic heart disease, similar to those observed in a number of large national and international cohorts of radiation oncologists.
	De Vocht, F. et al. 2021 [32]	Epidemiological study	The study performed sensitivity and probabilistic bias analyses to assess whether the association between external radiation exposure and ischemic heart disease mortality is robust to several assumptions made in the original matched case–control analyses.	The analyses conducted in this study provide further support to the hypothesis that the association observed between exposure to external radiation and mortality from ischemic heart disease may be causal.
	Kreuzer, M. et al. 2015 [33]	Epidemiological study	This study aims to examine exposure-response relationships between ionizing radiation and several mortality outcomes in a subgroup of 4054 men of the German uranium miner cohort study, who worked between 1946 and 1989 in milling facilities, but never underground or in open pit mines.	This study shows an excess mortality from lung cancer due to radon exposure and from solid cancers due to external gamma radiation in uranium millers that was not statistically significant. Exposure to uranium was not associated with any cause of death, but absorbed organ doses were estimated to be low.
**Diagnostically exposed groups**	Kitahara, C.M. et al. 2015 [34]	Epidemiological study	This study estimates risks of incidence and mortality from cancer and circulatory disease associated with performing procedures involving the use of radionuclides.	Performing radionuclide procedures was not associated with risks for most end points examined. The modest health risks among radiologic technologists performing procedures using radionuclides require further examination in studies with individual dose estimates, more detailed information regarding types of procedures performed and radionuclides used, and longer follow up.

## Data Availability

Not applicable.
